# Supplementary figures and images for: Cross-species transmission and PB2 mammalian adaptations of highly pathogenic avian influenza A/H5N1 viruses in Chile
Source: bioRxiv. 2023 Jun 30:2023.06.30.547205. Preprint. [Version 1] doi: 10.1101/2023.06.30.547205 (PMC10541606; doi:10.1101/2023.06.30.547205)

678 **Supplementary Figure 1: Map of Chile indicating HPAIV-positive cases by region between 12-**  
679 **toco09-2022 to 03-14-2023.**

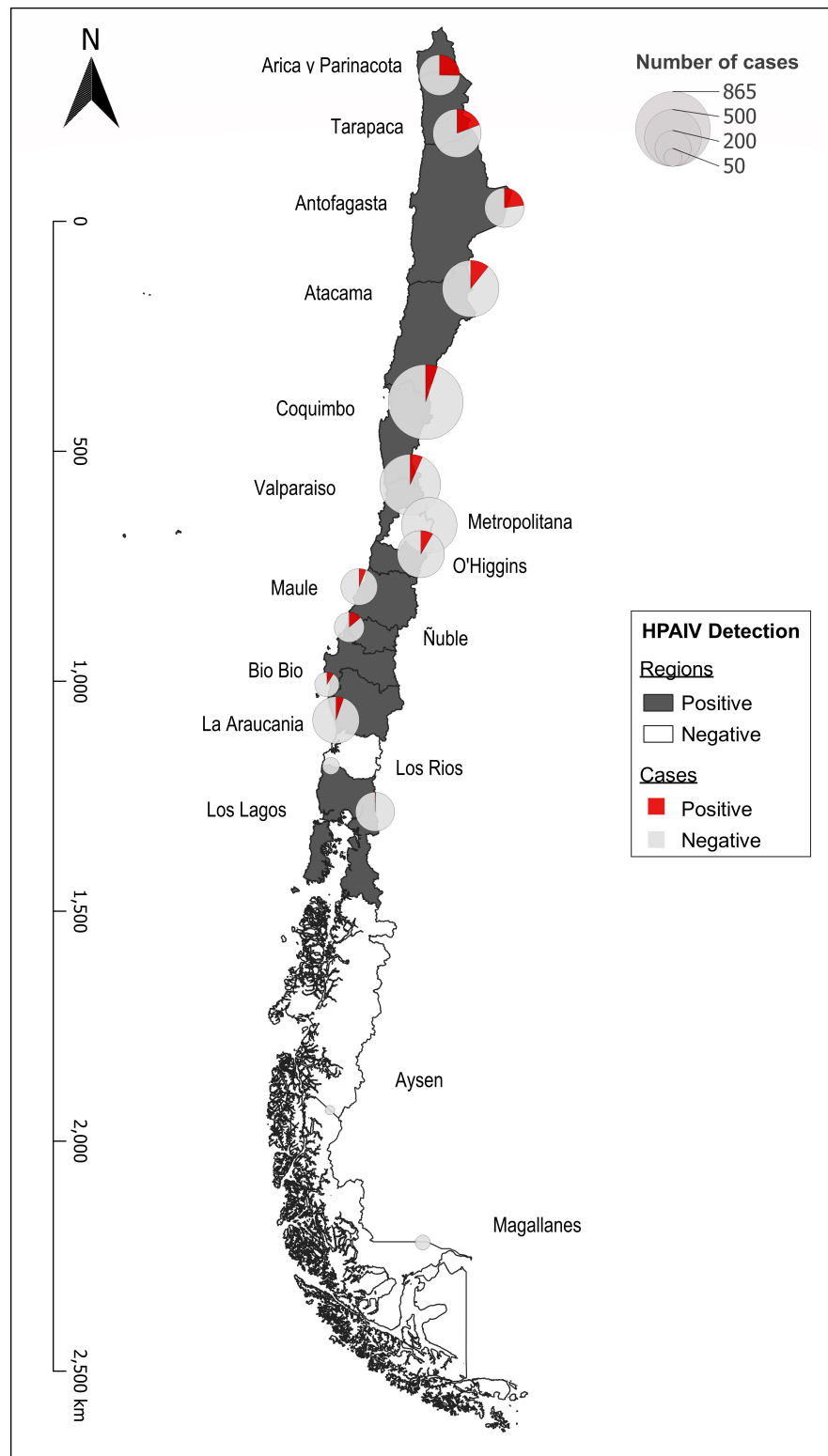

Supplement: 1 [file NIHPP2023.06.30.547205V1-supplement-1.pdf]
